# Molecular Mechanisms of Anti-Inflammatory Phytochemicals 2.0

**DOI:** 10.3390/ijms242417443

**Published:** 2023-12-13

**Authors:** Natália Cruz-Martins

**Affiliations:** 1 Faculty of Medicine, University of Porto, 4099-002 Porto, Portugal; ncmartins@med.up.pt; 2 Institute for Research and Innovation in Health (i3S), University of Porto, 4099-002 Porto, Portugal; 3 Institute of Research and Advanced Training in Health Sciences and Technologies (CESPU), Rua Central de Gandra, 4585-116 Gandra, Portugal; 4 TOXRUN—Toxicology Research Unit, University Institute of Health Sciences, CESPU, CRL, 4585-116 Gandra, Portugal

Inflammation is currently the most investigated cell response, not only for the frequency with which it occurs but essentially due to the growing incidence of inflammatory diseases, increasingly labeled as characteristics of modern society [1,2]. Despite multiple actors in this process, oxidative stress has been pointed out as the main factor responsible for the growing number and severity of the inflammatory diseases currently diagnosed and targeted by increasingly specific treatments [2,3]. Behind the oxidative stress, which is a common process, even due to the organism’s normal metabolism, an exaggerated production of free radicals is in its genesis, mostly triggered by an imbalance between reactive oxygen species (ROS) production and its elimination. In this process, and as a result of the lifestyle of modern society, the depletion of endogenous antioxidants is conceived as the key to the growing incidence of inflammatory disorders [2,4]. Capable of neutralizing the adverse effects of these ROS, endogenous antioxidants are increasingly deficient, even in individuals with a healthy lifestyle. Therefore, it is urgent to understand the molecular mechanisms behind the onset of such inflammatory diseases, of which cancer, neurodegenerative, and cardiovascular diseases are the most frequent.

On the other hand, and despite the morbimortality associated with such pathologies [5,6], currently available pharmacological treatments have been increasingly investigated, not only due to the increasing number of adverse effects and even toxicity reported, coupled with their ineffectiveness in multiple cases. Thus, not only the molecular mechanisms behind their pharmacological effects but also the mechanisms underlying the appearance of such clinical affections have been widely underlined.

As a result of the need to develop new drugs that are highly effective and minimally harmful to the body, the interest in researching and understanding the mechanisms of action of multiple bioactives present in plants and natural products, in general, has surprisingly reignited. Owing to their wide chemical composition, they have stood out from the beginning for their excellent antioxidant and anti-inflammatory activity, that is, in this case, extremely useful not only to modulate oxidative stress and assist endogenous antioxidant systems but also for jointly with currently available pharmacological therapies to increase its effectiveness and reduce the likelihood of adverse effects/toxicity occurrence. Thus, the second edition of this Special Issue (SI) entitled “Molecular mechanisms of anti-inflammatory phytochemicals 2.0” aims to present an updated perspective on naturally occurring bioactives currently under study for anti-inflammatory purposes and acclaim their mechanism of action.

In this SI, a total of six articles were published: four original works and two review articles. A brief overview of each published work is presented in the following paragraphs. In the first contribution, He and colleagues addressed in vivo the anti-inflammatory and hypolipidemic effects of a *Curcuma longa*-derived phytochemical, bisacurone. As the main findings, the authors stated that the less studied bioactive compound compared to curcumin, bisacurone, was capable of decreasing liver weight, blood viscosity, and the serum levels of cholesterol and triglycerides in mice receiving the high-fat diet. Similarly, mice receiving bisacurone evidenced lower levels of pro-inflammatory cytokines [namely interleukin-6 (IL-6) and tumor necrosis factor α (TNF-α)], and a marked inhibition of the phosphorylation of IKKα/β and NF-κB p65 subunit was also stated.

In the second contribution, Vaamonde-García and colleagues have investigated through an in vitro experiment, the effects of brown crude fucoidans in models of osteoarthritis; for such purpose, primary chondrocytes and the 260TT human chondrocyte cell line were used. Among other effects, a marked reduction in IL-6 levels was noted, along with an upregulation of Nrf-2 levels and the expression of the transcriptional genes, heme oxygenase-1 (HO-1) and superoxide dismutase 2 (SOD-2), both of extreme relevance for endogenous antioxidant systems.

The following two contributions, Qureshi and colleagues and Goc and colleagues, respectively, aimed to understand the mechanisms of action of soybean lectin and resveratrol and gallic acid, specifically in reprograming the gene expression of key inflammatory pathways in human peripheral blood mononuclear cells and in inhibiting *Borrelia burgdorferi*-induced TLR2-NF-kB signaling pathway, respectively. As the main findings stated by the authors in these works, resveratrol was proposed as a proteasome inhibitor and soybean lectins as a proteasome activator, with both being labeled as excellent cytokine expression modulators and mediators of expression in multiple signaling pathways linked to inflammation, being, thus, proposed as excellent candidates for preventing or even reversing inflammation-linked disorders. On the other hand, for gallic acid, starting with a minimum dose of 100 μg/mL, a pronounced anti-*B. burgdorferi* activity was noted, with this compound being also capable of inhibiting the cytokines secretion, particularly IL1β, IL6, and TNF-α.

Taken together, such experiments, despite underlining the need for future studies increasingly deepen, open the window of opportunities for developing upcoming anti-inflammatory drugs highly effective and less harmful to the human body while also constituting the cornerstone for designing proper review articles capable of summarizing all the literature available in a particular period related to a certain substance or even molecular pathway, in which the most effective compounds can be highlighted. In this SI, two review articles were published, one underlining the preventive effects on the skin of pharmaceutical phytochemicals targeting the Src family of tyrosine kinases and the Aryl hydrocarbon receptor (contribution 5) and the second one highlighting the in vivo evidence of the therapeutic potential of *Zingiber officinale* Rosc. in metabolic syndrome (contribution 6). In the first review article, the most promisor phytochemicals discussed were 4-phynylpyrdine, withanolides, malic and isocitric acid, myricetin, quercetin, caffeic acid, cryptotanshinone, ursolic acid, curcumin, baicalein, 5-deoxykaempferol, diosmin, cinnamaldehyde, cynaropicrin, baicalein, kaempferol, pterostilbene, and oleanolic acid. For all, very interesting anti-inflammatory effects were stated, acting at different levels and thus all improving skin health, mostly through topical application. In the second review article, the authors briefly discussed the therapeutic potential of ginger and their derived bioactives in cardiovascular diseases, obesity, diabetes mellitus, non-alcoholic fatty liver disease, alcohol addiction, and prostate complications.

In short, all data published in this SI, from original to review articles highlight the real importance of addressing the multiple potentialities of naturally occurring bioactives for therapeutic purposes. Used since ancient times, such molecules present a plethora of potentialities, and unlike chemical substances, they mostly act by modulating a specific pathway and not blocking or activating; it is also interesting to note that they normally act in a multi-away, which means that using a certain substance we can achieve several benefits in various organs, systems or even tissues, culminating in the promotion of health and wellbeing and prevention or even treatment of a disease.

## References

[1] Aleksandrova K., Koelman L., Rodrigues C.E. (2021). Dietary patterns and biomarkers of oxidative stress and inflammation: A systematic review of observational and intervention studies. Redox Biol..

[2] Forman H.J., Zhang H. (2021). Targeting oxidative stress in disease: Promise and limitations of antioxidant therapy. Nat. Rev. Drug Discov..

[3] Yilmaz M.I., Romano M., Basarali M.K., Elzagallaai A., Karaman M., Demir Z., Demir M.F., Akcay F., Seyrek M., Haksever N. (2020). The Effect of Corrected Inflammation, Oxidative Stress and Endothelial Dysfunction on Fmd Levels in Patients with Selected Chronic Diseases: A Quasi-Experimental Study. Sci. Rep..

[4] Biswas S.K. (2016). Does the Interdependence between Oxidative Stress and Inflammation Explain the Antioxidant Paradox?. Oxid. Med. Cell. Longev..

[5] Armocida B., Monasta L., Sawyer S., Bustreo F., Segafredo G., Castelpietra G., Ronfani L., Pasovic M., Hay S., Armocida B. (2022). Burden of non-communicable diseases among adolescents aged 10–24 years in the EU, 1990–2019: A systematic analysis of the Global Burden of Diseases Study. Lancet Child Adolesc. Health.

[6] Tran K.B., Lang J.J., Compton K., Xu R., Acheson A.R., Henrikson H.J., Kocarnik J.M., Penberthy L., Aali A., Abbas Q. (2022). The global burden of cancer attributable to risk factors, 2010–2019: A systematic analysis for the Global Burden of Disease Study. Lancet.

